# Prognostic analysis of pT1-T2aN0M0 cervical adenocarcinoma based on random survival forest analysis and the generation of a predictive nomogram

**DOI:** 10.3389/fonc.2022.1049097

**Published:** 2022-11-24

**Authors:** Dong Ouyang, Mengting Shi, Yiman Wang, Limin Luo, Luzhong Huang

**Affiliations:** ^1^ Department of Obstetrics and Gynecology, Taizhou Women and Children’s Hospital of Wenzhou Medical University, Taizhou, Zhejiang, China; ^2^ Department of Textile Engineering, Akesu Regional Vocational and Technical College, Akesu, Xinjiang, China; ^3^ Department of Pathology, Taizhou Women and Children’s Hospital of Wenzhou Medical University, Taizhou, Zhejiang, China

**Keywords:** adjuvant treatment, prognosis, cervical adenocarcinoma, random survival forest, nomogram, SEER, conditional survival forest

## Abstract

**Background:**

The efficacy of adjuvant radiotherapy for postoperative patients with early-stage cervical adenocarcinoma who are lymph node-negative is still inconclusive. Establishing a nomogram to predict the prognosis of such patients could facilitate clinical decision-making.

**Methods:**

We recruited 4636 eligible patients with pT1-T2aN0M0 cervical adenocarcinoma between 2004 and 2016 from the Surveillance, Epidemiology and End Results (SEER) database. Random survival forest (RSF) and conditional survival forest (CSF) model was used to assess the prognostic importance of each clinical characteristic variable. We identified independent prognostic factors associated with overall survival (OS) by univariate and multivariate Cox regression risk methods and then constructed a nomogram. We stratified patients based on nomogram risk scores and evaluated the survival benefit of different adjuvant therapies. To reduce confounding bias, we also used propensity score matching (PSM) to match the cohorts before performing survival analyses.

**Results:**

The RSF and CSF model identified several important variables that are associated with prognosis, including grade, age, radiotherapy and tumor size. Patients were randomly divided into training and validation groups at a ratio of 7:3. Multivariate cox analysis revealed that age, grade, tumor size, race, radiotherapy and histology were independent prognostic factors for overall survival. Using these variables, we then constructed a predictive nomogram. The C-index value for evaluating the prognostic nomogram fluctuated between 0.75 and 0.91. Patients were divided into three subgroups based on risk scores, and Kaplan-Meier (K-M) survival analysis revealed that in the low-risk group, postoperative chemotherapy alone was associated with a significantly worse OS than surgery alone. Following PSM, survival analysis showed that compared with surgery alone, radiotherapy was associated with a worse OS in the training group although there was no significant difference in the validation group.

**Conclusions:**

For patients with pT1-T2aN0M0 cervical adenocarcinoma, adjuvant treatments such as postoperative radiotherapy or chemotherapy, compared with surgery alone, are of no benefit with regards to patient survival. Our prognostic nomogram exhibits high accuracy for predicting the survival of patients with early-stage postoperative cervical adenocarcinoma.

## Introduction

Cervical cancer is one of the most common malignancies of the female reproductive tract and the fourth leading cause of cancer-related death in women. An estimated 341,831 people worldwide were predicted to die from this form of cancer in 2020 (1). Cervical cancer mainly originates from the squamous epithelium and glandular epithelium of the cervix, of which adenocarcinoma accounts for approximately 25% (2, 3). In Western countries, such as the United Kingdom and the United States, the incidence of cervical adenocarcinoma is gradually increasing (3, 4). The prognosis of patients with cervical adenocarcinoma is significantly worse than that of patients with squamous cell carcinoma, furthermore, these patients are prone to distant metastasis. Furthermore, the pathological type of adenocarcinoma is an independent risk factor for the poor prognosis of patients with cervical cancer (5–8).

For locally advanced cervical carcinoma, there is a consensus that whole-pelvic radiation therapy has the best outcome when combined with cisplatin-based chemotherapy (9). The best treatment for earlier stage cervical carcinoma is optimized surgery (9–12). Postoperative high-risk factors for early cervical cancer include lymph node metastasis, parametrial invasion and positive surgical margins. The American Society for Radiation Oncology (ASTRO) guidelines propose that patients with high-risk factors after cervical cancer surgery should receive postoperative supplemental pelvic radiotherapy combined with cisplatin chemotherapy (9). However, in patients with intermediate risk factors after surgery, such as tumor size, pathological type and tumor differentiation, there is no conclusive evidence of the need for radiotherapy and chemotherapy. In a previous study, Mabuchi considered adenocarcinoma to be an independent prognostic indicator for poor survival in patients with early-stage cervical cancer with both intermediate and high-risk factors, regardless of the type of adjuvant radiotherapy after radical hysterectomy (13). In another study, Ryud et al. considered that adenocarcinoma represents a moderate risk factor and that whole pelvic radiotherapy should be performed following surgery (14). Zhou et al. suggested that for patients with lymph node-positive early-stage cervical cancer, cervical adenocarcinoma had a significantly worse survival rate than cervical squamous cell carcinoma that could benefit from concurrent chemoradiotherapy (CCRT); however, patients with adenocarcinoma did not benefit from CCRT (15).

However, some researchers believe that the addition of CCRT after radical hysterectomy does not significantly improve the survival of patients with pelvic lymph node-positive FIGO stage IIIC1 cervical adenocarcinoma (16). For example, Mahmoud et al. concluded that adjuvant chemoradiotherapy did not have a significant survival advantage over adjuvant radiotherapy in postoperative women with early-stage cervical cancer with intermediate risk factors (17). Compared with cervical squamous cell carcinoma, cervical adenocarcinoma exhibits obvious heterogeneity in terms of tissue anatomy, prognosis and recurrence, although current treatment is unable to distinguish cervical adenocarcinoma. At present, there is no evidence to indicate which clinicopathological risk factors affect the prognosis of lymph node-negative and non-metastatic early-stage cervical adenocarcinoma after surgery or whether these patients can benefit from adjuvant therapies such as postoperative radiotherapy and chemotherapy.

Large-scale clinical trials and studies are restricted due to the low incidence of cervical adenocarcinoma. The Surveillance, Epidemiology and End Results (SEER) database is a nationwide cancer dataset compiled in the United States and features a wealth of relevant information about patients with different types of cancer, thus providing appropriate real-world population data for our research. In this study, we used RSF and CSF model to evaluate the importance of clinicopathological variables in the prognosis of cervical adenocarcinoma patients identified in the SEER database. Then, we constructed a predictor nomogram of overall survival (OS) for patients with pT1-T2aN0M0 after surgery to facilitate clinical decision making. In addition, we also investigated whether postoperative adjuvant chemoradiotherapy can benefit early cervical adenocarcinoma after surgery.

## Methods

### Data collection

**Figure 1:**
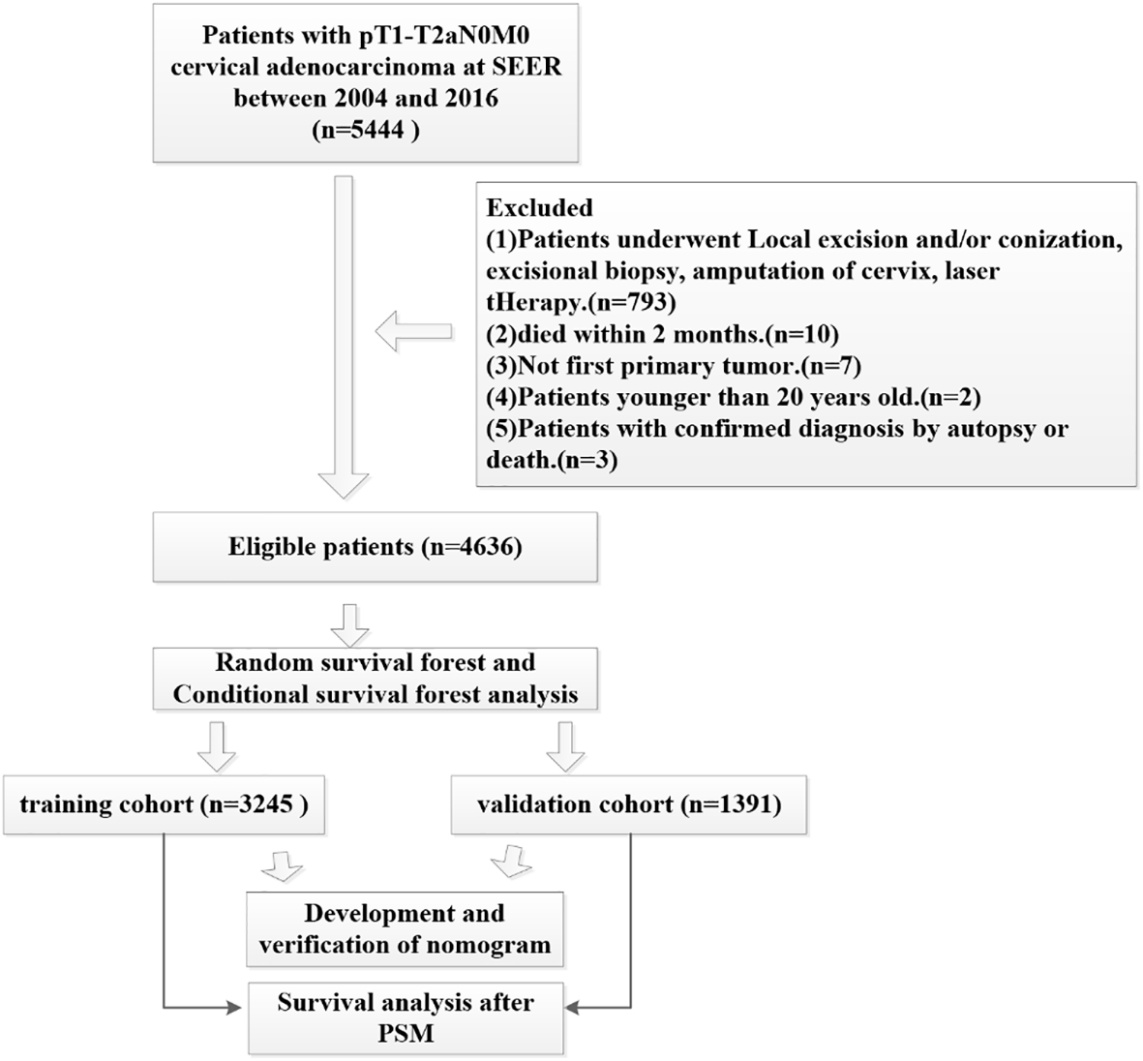
Flow chart depicting the design of this study.

This study analyzed data from the publicly available SEER database from 2004 to 2016. We extracted data using SEER*Stat Software Version 8.3.9.2 (https://seer.cancer.gov/data-software/) with a permitted SEER ID (19968-Nov2020). Because this data is open access, no ethical approval was required. Our study cohort applied that following inclusion criteria (1): All patients underwent hysterectomy and radical hysterectomy (surgery encode: 30-70) (2); none of the patients had lymph node metastasis or distant metastasis (3); cervical adenocarcinoma was the first primary tumor (4); the primary sites for all patients were C53.0-endocervix, C53.1-exocervix, C53.9-cervix uteri and C53.8-overlapping lesions of cervix uteri (5); all patients showed histology codes 8140 to 8389 with adenomas and adenocarcinomas based on ICD-O-3 (6); according to the ACJJ 8, T staging was T1-T2a, and (7) complete follow-up data was available. The exclusion criteria were as follows (1): patients younger than 20 years-of-age (2); all patients who died within 2 months were excluded (3); patients undergoing local excision and/or conization, excisional biopsy, amputation of the cervix or those undergoing laser therapy (4), not first primary tumor, and (5) patients with a confirmed diagnosis by autopsy or death. We used the random forest method to replace missing data with multiple imputation; a specific flowchart describing patient recruitment is given in **Figure 1**.

We extracted the following demographic and clinicopathological characteristics for analysis: age at diagnosis, marital status, histological grade, race, histopathological type, surgery, AJCC Seventh T stage, tumor size, chemotherapy and radiotherapy. Overall survival (OS) was used to evaluate patient prognosis. The death outcome for OS was death by any cause.

### Random survival forest analysis

Random survival forest (RSF) analysis is an ensemble machine learning method that adds survival analysis to random forest analysis (18). This strategy is bound by the hazard proportionality assumption and the log linearity assumption. In addition, RSF can also rank the importance of variables according to the minimal depth of the largest subtree in which the variable is located. In this cohort, we adopted the random forest algorithm to fill in some missing values. We also explored the prognostic importance of clinical feature variables in early-stage cervical adenocarcinoma using RSF models.

### Conditional survival forest analysis

Disadvantages of RSF include a bias towards inclusion of variables with many split points (19–21). This effect leads to deviations in the estimates of the summary of results, such as variable importance. And conditional inference forest (CIF) can reduce this selection bias by separating the algorithm for selecting the best covariate to be segmented from the algorithm for searching the best split point. Therefore, CSF can select variables according to the importance of variables, and then select the split area. We then used the CSF model to explore the importance of clinical feature variables.

### Nomogram construction

We screened eligible patients for inclusion in our study. Then, these patients were randomly assigned to training and validation groups in a 7:3 ratio. A nomogram was subsequently constructed and validated with the Cox method. Cox proportional hazards regression was then used to identify independent prognostic factors. Based on the variables screened by Cox multivariate analysis, a nomogram was established to predict the prognosis of patients with early-stage cervical cancer. The concordance index (C-index), receiver operating characteristic (ROC) curve and calibration curves were used to evaluate the robustness of the nomogram. Based on the nomogram, we then calculated a risk score for each patient. The optimal cut-off value for patient risk scores was determined using X-tile software (https://medicine.yale.edu/lab/rimm/research/software/) and patients were divided into three risk subgroups.

### Statistical analysis

All analyses were performed using R statistical software (http://www.r-project.org, version 3.6.1). Clinicopathological baseline characteristics of patients were described using proportions and frequencies, and comparisons between different subgroups were performed using Fisher’s exact test. Kaplan-Meier (K-M) survival curves were evaluated using the log-rank test. The proportional risk hypothesis for each covariate included in the Cox model fitting was tested. Hazard ratio (HR) values were calculated using Cox proportional hazard regression models. Variables with a P value <0.1 in the Cox univariate analysis were then included in Cox multivariate analysis. To reduce selection bias due to unbalanced perioperative factors, propensity score matching (PSM) was used in the analyses of non-radiotherapy and radiotherapy groups. We use the “nearest neighbor method” method for matching according to a ratio of 1:1. A two-sided P<0.05 was considered to be statistically significant. The CSF model was analyzed using “PySurvival” in Python version 3.6 (Python Software Foundation for Statistical Computing, Wilmington, Delaware, USA). All other analyses were performed using packages in the R software (http://www.r-project.org, version 3.6.1), including “tableone”, “randomForestSRC”, “survival”, “survminer”, “rms”, “Cairo”, “pec” and “MatchIt”.

## Results

### Patient characteristics

**Figure 2:**
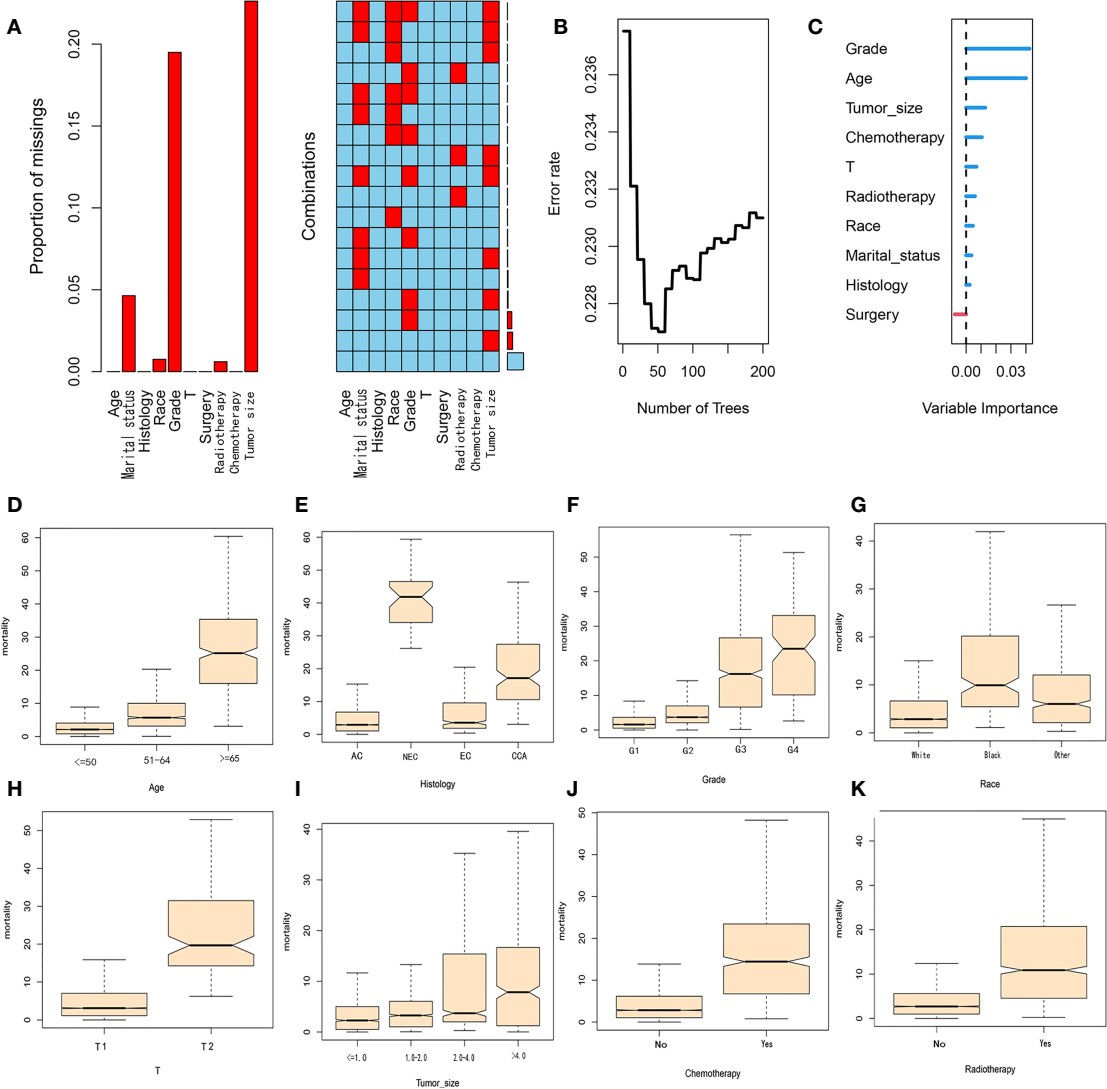
**(A)** Proportion and combination of missing values in the cohort. **(B)** Random forest plot. **(C)** Variable importance score. **(D–K)** The contribution of variables in each subgroup to mortality.

A total of 4636 patients diagnosed with pT1-2aN0M0 cervical adenocarcinoma were identified in the SEER database and met our inclusion criteria. Of these patients, 3953 were white and 3278 were younger than 50 years-of-age, with a mean follow-up of 62 months (range: 2 - 155 months). In our cohort, there were many missing values for key variables such as tumor size and grade (see **Figure 2A**). To avoid reducing the robustness of our model, we used a random forest method to fill in these missing values. This is a common method in machine learning and represents an ensemble learning model based on decision tree classifiers. We implemented random forest filling using the misforest function in the misforest package in R. Subsequently, patients were randomly divided into training (n=3245) and validation (n=1391) sets at a ratio of 7:3. Detailed baseline demographic and clinical characteristics are summarized in **Table 1**. There were no significant differences in the demographic and clinicopathological characteristics when compared between the training and validation sets. The OS rates at 1, 3 and 5 years were 99.0%, 95.6% and 92.7% in the training cohort and 98.9%, 96.3% and 93.7% in the validation cohort, respectively.

**Table 1:** Demographics and clinicopathological characteristics of patients with pT1-T2aN0M0 cervical adenocarcinoma.

Characteristics	Whole cohort	Training cohort	Validation cohort	P value
Overall No.	4636	3245	1391	0.771
Age (years)				
<=50	3278 (70.7)	2304 (71.0)	974 (70.0)	
51-64	978 (21.1)	676 (20.8)	302 (21.7)	
>=65	380 (8.2)	265 (8.2)	115 (8.3)	
Histology				0.464
AC	4064 (87.7)	2851 (87.9)	1213 (87.2)	
NEC	41 (0.9)	25 (0.8)	16 (1.2)	
EC	403 (8.7)	284 (8.8)	119 (8.6)	
CCA	128 (2.8)	85 (2.6)	43 (3.1)	
T				0.78
T1	4511 (97.3)	3162 (97.4)	1349 (97.0)	
T2a	125 (2.7)	83 (2.6)	42 (3.0)	
Race (%)				0.762
White	3953 (85.3)	2761 (85.1)	1192 (85.7)	
Black	220 (4.7)	159 (4.9)	61 (4.4)	
Other	463 (10.0)	325 (10.0)	138 (9.9)	
Grade (%)				0.196
Well-differentiated	2088 (45.0)	1484 (45.7)	604 (43.4)	
Moderately differentiated	1766 (38.1)	1236 (38.1)	530 (38.1)	
Poorly differentiated	687 (14.8)	464 (14.3)	223 (16.0)	
Undifferentiated	95(2.0)	61 (1.9)	34 (2.4)	
Surgery				0.1788
Total hysterectomy	2375 (51.2)	1641 (50.6)	734 (52.8)	
Radical hysterectomy	2261 (48.8)	1604 (49.4)	657 (47.2)	
Radiotherapy				
No	3753 (81.0)	2607 (80.3)	1146(82.4)	
Yes	883 (19.0)	638 (19.7)	245 (17.6)	
Chemotherapy				0.547
No	4101 (88.5)	2864 (88.3)	1237(88.9)	
Yes	535 (11.5)	381 (11.7)	154 (11.1)	
Tumor size (cm)				
<=1.0	1597 (34.4)	1102 (34.0)	495 (35.6)	
1.0-2.0	1393 (30.0)	986 (30.4)	407 (29.3)	
2.0-4.0	1263 (27.2)	887 (27.3)	376 (27.0)	
>4.0	383 (8.3)	270 (8.3)	113 (8.1)	

AC, Adenocarcinoma; NECC, Neuroendocrine carcinoma; CCA, Clear cell adenocarcinoma; EC, Endometrioid carcinoma.

### RSF and CSF analysis

**Figure 3:**
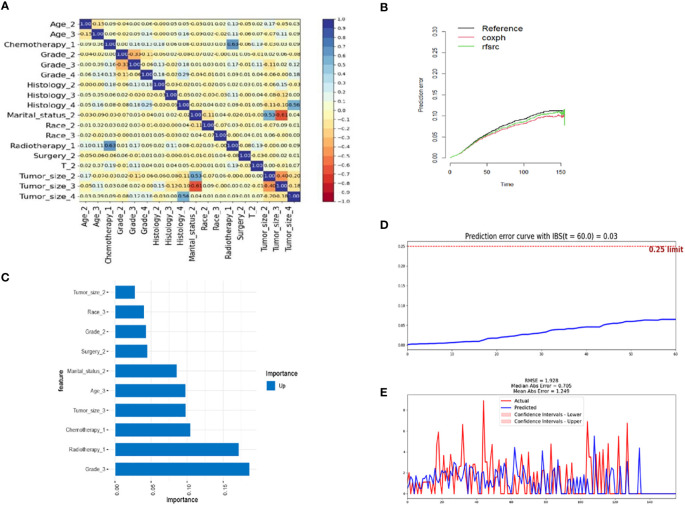
Conditional survival forest analysis **(A)** Correlation of clinical feature variables, **(B)** Prediction errors of Cox regression model and RSF model, **(C)** Variable importance score, **(D)** Prediction error curve with Integrated Brier Score (IBS), **(E)** Number of dead patients.

The key advantage of the random survival forest model is that it is not constrained by the proportional hazard assumption and the logarithmic linear assumption. Furthermore, RSF analysis can avoid the overfitting problem associated with some other algorithms by applying two random sampling processes (22). We trained a RSF with 200 trees in the entire dataset with a terminal node size of 15 and randomly selected mtry=3 variables per iteration. RSF analysis uses Harrell’s concordance index to calculate accuracy. From **Figure 2B**, it can be concluded that the C-index of the model is approximately 0.77 and when the survival tree increased to a certain number, the error rate curve tended to be stable (**Figure 2B**). The variable importance of predicting patient prognosis is demonstrated in **Figure 2C**. The variable importance score, obtained by the minimum depth method, was determined to be grade, age, tumor-size, chemotherapy and T-stage (from high to low). The contribution of each subgroup variable to mortality is shown in **Figures 2D–K**. Subgroup variables such as age (>=65 years), NEC, G3, G4 and T2 contributed the most to mortality. In the CSF model, we used 20 minimum node sizes, 200 trees, and a 0.05 alpha. We calculated the correlation between clinical variables, and the results are shown in **Figure 3A**. Subsequently, we compared the prediction errors of COX regression model and RSF model based on brief score. As shown in **Figure 3B**, both have similar prediction performance. We calculated the patient’s risk factors using the CSF model (**Figure 3C**), The order of importance of variables is grade, Radiotherapy, Chemotherapy, Tumor Size, age, etc. We also tested the predictive errors of the model. The results showed that when the time was 60 months, the Brief score was 0.03 (**Figure 3D**). The comparison between the overall prediction and the actual number of deaths is shown in the **Figure 3E**. The above show that the CSF model has good prediction ability.

### Prognostic analysis of patients with pT1-2aN0M0 cervical adenocarcinoma

**Figure 4:**
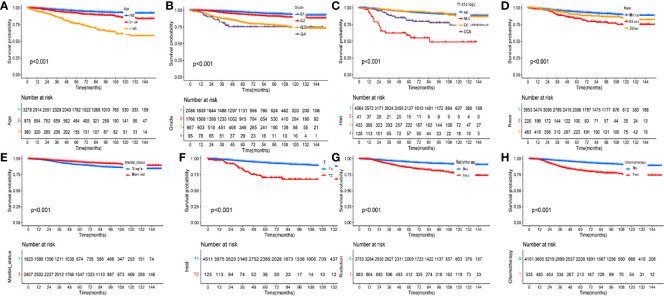
Kaplan-Meier curves comparing overall survival (OS) for pT1-T2aN0M0 cervical adenocarcinoma patients stratified by **(A)** age, **(B)** grade, **(C)** histology, **(D)** race, **(E)** marital status, **(F)** T stage, **(G)** radiotherapy and **(H)** chemotherapy, respectively.

Kaplan-Meier curves were used to compare the OS of patients with pT1-T2aN0M0 cervical adenocarcinoma, as stratified by clinical variables; the results are shown in **Figures 4A–H**. Patients in the radiotherapy or chemotherapy groups had significantly worse survival outcomes when compared to the non-radiotherapy or non-chemotherapy groups (P < 0.001; **Figures 4G, H**). We used univariate and multivariate analyses to further explore the prognostic factors affecting the training cohort of patients with cervical adenocarcinoma identified from the SEER database. **Table 2** shows results for the training cohort. Each covariate included in the Cox model fitting conforms to the proportional risk assumption. Cox multivariate hazard ratio analysis showed that several variables were independent prognostic risk factors for overall survival, including age at diagnosis, race, grade, histology, tumor size and radiotherapy

**Table 2:** Univariate and multivariate analysis for the prognostic characteristics of OS.

Characteristics	Univariate analysis			Multivariate analysis		
	HR	95%CI	P value	HR	95%CI	P value
Age (years)						
<=50	Ref			Ref		
51-64	2.11	1.54-2.91	0	1.74	1.25-2.44	0.0011
>=65	6.59	4.84-8.98	0	4.36	3.09-6.16	0
Marital status						
Single	Ref			Ref		
Married	0.62	0.48-0.8	0	0.74	0.51-1.09	0.1254
Histology						
AC	Ref			Ref		
NEC	10.21	5.69-18.33	0	4.38	2.23-8.62	0
EC	1.03	0.65-1.63	0.907	0.93	0.56-1.53	0.7752
CCA	2.46	1.4-4.33	0.002	0.56	0.26-1.25	0.1574
Race (%)						
White	Ref			Ref		
Black	2.4	1.55-3.7	0	2.13	1.36-3.33	9.00E-04
Other	1.43	0.96-2.12	0.081	1.49	0.99-2.22	0.0542
T						
T1	Ref			Ref		
T2	3.37	2-5.69	0	1.28	0.74-2.22	0.3824
Grade (%)						
Well-differentiated	Ref			Ref		
Moderately differentiated	2.03	1.42-2.89	0	1.87	1.31-2.68	6.00E-04
Poorly differentiated	6.08	4.28-8.64	0	4.51	3.09-6.6	0
Undifferentiated	9.98	5.4-18.41	0	4.64	2.4-8.97	0
Surgery						
Total hysterectomy	Ref			Ref		
Radical hysterectomy	0.76	0.59-0.99	0.043	0.88	0.68-1.16	0.3729
Radiotherapy						
No	Ref			Ref		
Yes	2.89	2.22-3.77	0	1.78	1.26-2.51	0.001
Chemotherapy						
No	Ref			Ref		
Yes	3.07	2.28-4.14	0	1.15	0.78-1.68	0.485
Tumor size (cm)						
<=1.0	Ref			Ref		
1.0-2.0	1.54	1.03-2.29	0.033	1.68	1.05 - 2.71	0.0322
2.0-4.0	2.8	1.93-4.06	0	1.52	0.99 - 2.33	0.0555
>4.0	2.7	1.67-4.37	0	1.52	0.83 - 2.81	0.1785

AC, Adenocarcinoma; NECC, Neuroendocrine carcinoma; CCA, Clear cell adenocarcinoma; EC, Endometrioid carcinoma.

### Construction and validation of a predictive nomogram

**Figure 5:**
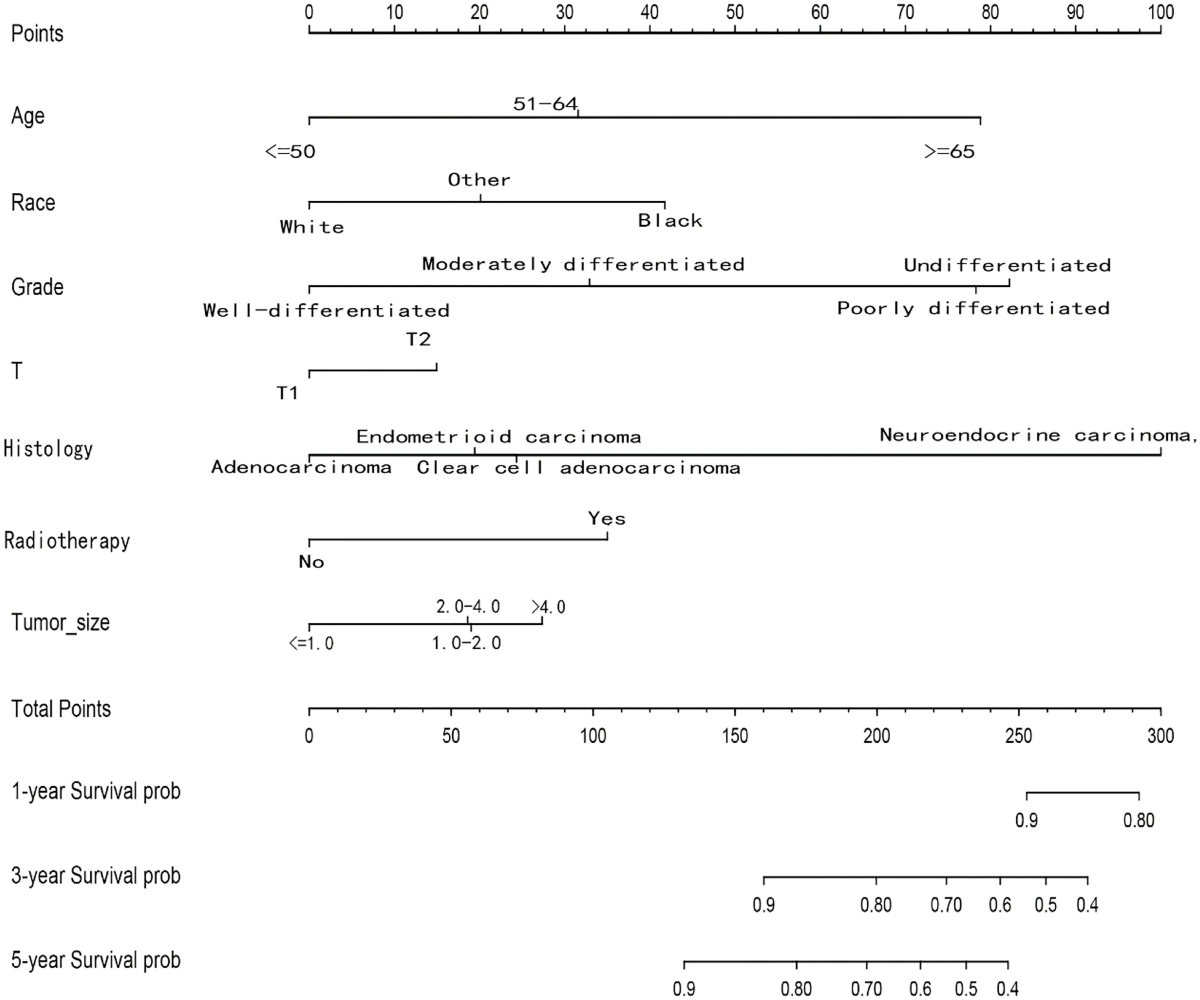
Nomograms for predicting the 1-, 3- and 5-year OS rates of patients with pT1-T2aN0M0 cervical adenocarcinoma undergoing surgery.

**Figure 6:**
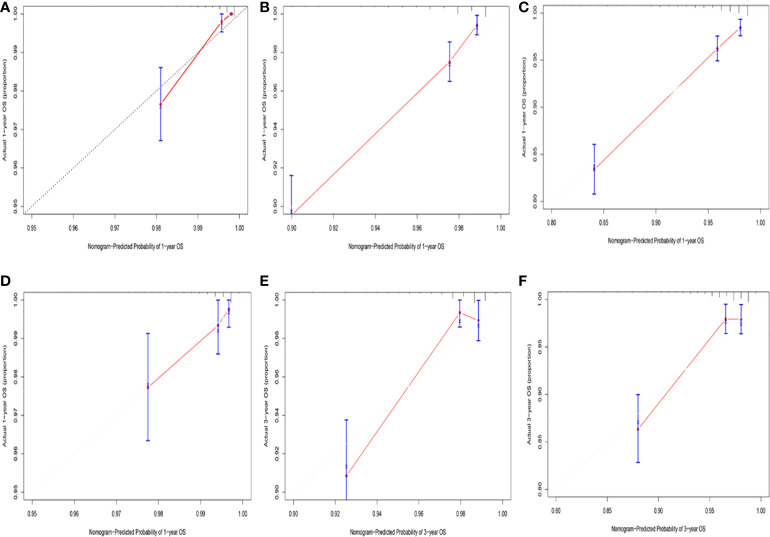
**(A)** Calibration curve for predicting 1-year OS rates in the training cohort. **(B)** Calibration curve for predicting 3-year OS rates in the training cohort. **(C)** Calibration curve for predicting 5-year OS rates in the training cohort. **(D)** Calibration curve for predicting 1-year OS rates in the validation cohort. **(E)** Calibration curve for predicting 3-year OS rates in the validation cohort. **(F)** Calibration curve for predicting 5-year OS rates in the validation cohort.

**Figure 7:**
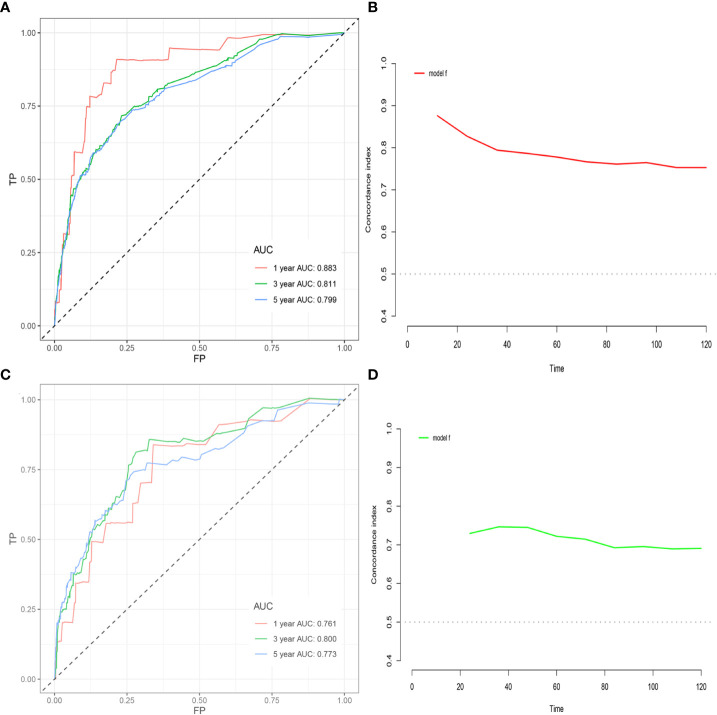
**(A)** ROC curve based on the nomogram for the training cohort. **(B)** C-index value based on nomogram in the training cohort. **(C)** ROC curve based on nomogram in the validation cohort. **(D)** C-index value based on the nomogram in the validation cohort.

Among the variables identified by multivariate analysis, there were no T stage and chemotherapy. Combined with the screening of previous survival forest models, and considering the importance of T staging in clinical practice, we added T stage to the variables identified by cox multivariate analysis to be included in the predictive nomogram. Adjuvant therapy such as radiotherapy and chemotherapy will be further studied in the follow-up analysis. Based on these variables, we constructed a nomogram to assess 1-, 3- and 5-year survival in the training cohort with early-stage pT1-2aN0M0 cervical adenocarcinoma (**Figure 5**). The model assigned beta-coefficients to each of the included variables. In this manner, we created a linear equation and calculated each patient’s risk score to predict her prognosis. Then we verified the prediction nomogram in the verification cohort. Calibration curves for the training and validation groups showed that the survival rate, as predicted by the model, was consistent with actual observations (**Figures 6A–F**). ROC curves for the training set and validation group are shown in **Figures 7A, C**, respectively. The C-index of the model fluctuated between 0.75 and 0.91 in the training set and between 0.72 and 0.81 in the validation group (**Figures 7B, D**).

### Risk stratification according to the nomogram

**Figure 8:**
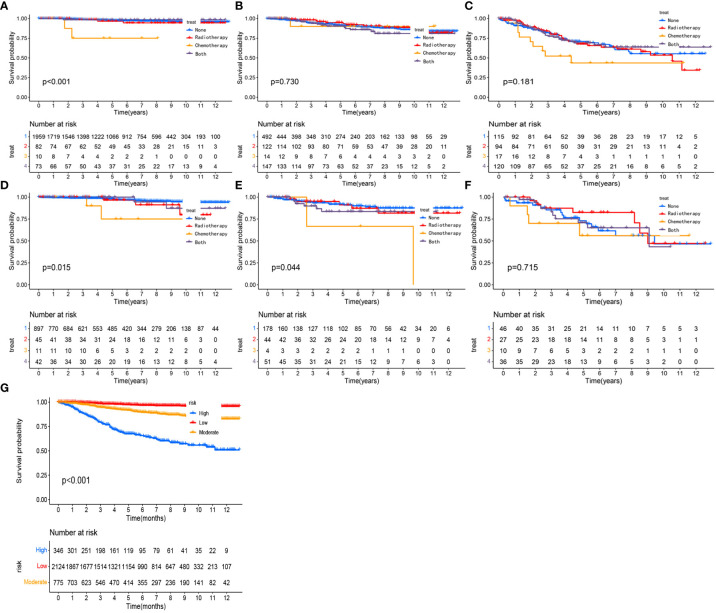
Kaplan-Meier curves comparing the survival effects of different adjuvant treatment strategies for patients with pT1-T2aN0M0 cervical adenocarcinoma in the low-risk training cohort **(A)**, moderate-risk training cohort **(B)**, the high-risk training cohort **(C)**, the low-risk validation cohort **(D)**, the moderate-risk validation cohort **(E)**, and the high-risk validation cohort **(F)**. Kaplan-Meier curves after the risk stratification of patients in the training cohort **(G)**.

We calculated a risk score for each patient based on the nomogram and then performed risk stratification analysis based on risk scores using X-tile software (23). Cut-offs were set at 0.25 and 1.30, respectively, with a risk score < 0.25 as a low-risk group, between 0.25 and 1.30 as a medium-risk group and > 1.30 as a high-risk group. Patients with pT1-2aN0M0 cervical adenocarcinoma could be divided into three sub-groups according to risk scores. In the training cohort, the 5-year OS decreased significantly with increasing risk and was 92.7%, 88.5% and 74.9% in the low, moderate and high-risk groups, respectively (p < 0.01). In the validation cohort, the 5-year OS rates were 97.6%, 89.4% and 71.6% in the low, moderate and high-risk groups, respectively (p < 0.001). As shown in **Figure 8G**, after risk stratification, the different groups of patients showed significant differences following K-M curve survival analysis.

### Subgroup analysis after risk stratification

To assess whether postoperative adjuvant therapies, such as radiotherapy and chemotherapy, could benefit survival in each subgroup, we plotted KM survival curves for different adjuvant treatments in each subgroup and then calculated hazard ratios for each adjuvant therapy. For patients in the low-risk training and validation cohorts, postoperative chemotherapy alone exhibited a significantly worse OS than surgery alone (**Figures 8A, D**, p<0.001). In the moderate risk validation group, postoperative chemotherapy alone had a worse OS when compared with surgery alone (**Figure 8E**, p=0.044). In the other groups, there was no significant difference in terms of OS when compared between surgery alone and other adjuvant therapies (**Figures 8B, C, F**). In each subgroup, the hazard ratios for each adjuvant therapy are detailed in **Table 3**.

**Table 3:** Univariate analysis evaluating the effect of adjuvant treatment strategies stratified by subgroups.

Adjuvant treatment	Low risk group		Moderate risk group		High risk group	
	HR (95% CI)	P value	HR (95% CI)	P value	HR (95% CI)	P value
Surgery alone	Ref		Ref		Ref	
RT	0.38 (0.45-4.69)	0.53	0.88 (0.46-1.70)	0.71	1.05 (0.64-1.72)	0.842
CT	2.66 (3.46-59.14)	<0.001	0.92 (0.13-6.68)	0.94	2.06 (0.98-4.32)	0.055
Both	0.50 (0.08-4.39)	0.62	1.33 (0.74-2.39)	0.34	0.92 (0.56-1.52)	0.757

RT, Radiotherapy; CT, Chemotherapy.

### Survival analysis after PSM

**Figure 9:**
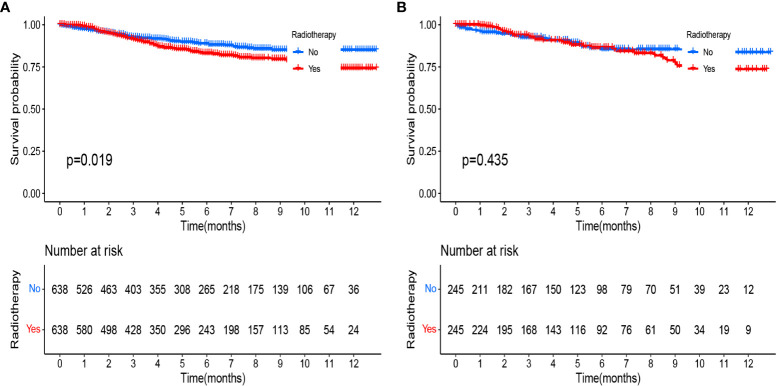
Kaplan-Meier curves comparing the survival effects of radiotherapy for patients with pT1-T2aN0M0 cervical adenocarcinoma after PSM **(A)** in the training cohort and **(B)** in the validation cohort.

Both our previous survival analysis and Cox multivariate analysis suggested that radiotherapy was a poor prognostic factor. Radiotherapy failed to improve prognosis; rather, it appeared to increase the poor survival outcomes of patients. When we analyzed different groups of radiotherapy patients, we found that high-risk groups were more likely to receive radiotherapy (**Tables 4**, **5**). To reduce bias, we used propensity score matching (PSM) for subsequent survival analyses. Patients in the training set and validation set were matched in a 1:1 ratio according to the PSM method, respectively (**Tables 4**, **5**). After matching patients 1:1 with PSM to control for confounding bias. In the training cohort, KM survival analysis revealed significantly worse survival in the radiotherapy group than in the non-radiotherapy group (**Figure 9A**, p=0.019). However, in the validation cohort, KM survival curves revealed no significant differences in terms of survival between the radiotherapy groups compared with the non-radiotherapy groups (**Figure 9B**, P >0.05).

**Table 4:** Baseline characteristics of patients with pT1-T2aN0M0 cervical adenocarcinoma in the training cohort.

Characteristic	No. of patients before PSM (%)			No. of patients after PSM (%)		
	No RT (n= 2607)	RT (n = 638)	P-value	No RT (n = 638)	RT (n= 638)	P-value
Age (years)			<0.001			0.867
<=50	1949 (74.8)	355 (55.6)		351 (55.0)	355 (55.6)	
51-64	477 (18.3)	199 (31.2)		207 (32.4)	199 (31.2)	
>=65	181 (6.9)	84 (13.2)		80 (12.5)	84 (13.2)	
Marital status			0.115			0.081
Single	1025 (39.3)	273 (42.8)		305 (47.8)	273 (42.8)	
Married	1582 (60.7)	365 (57.2)		333 (52.2)	365 (57.2)	
Histology			<0.001			0.833
AC	2339 (89.7)	512 (80.3)		506 (79.3)	512 (80.3)	
NEC	18 (0.7)	7 (1.1)		7 (1.1)	7 (1.1)	
EC	191 (7.3)	93 (14.6)		92 (14.4)	93 (14.6)	
CCA	59 (2.3)	26 (4.1)		33 (5.2)	26 (4.1)	
Race (%)			0.281			0.508
White	2226 (85.4)	535 (83.9)		533 (83.5)	535 (83.9)	
Black	120 (4.6)	39 (6.1)		48 (7.5)	39 (6.1)	
Other	261 (10.0)	64 (10.0)		57 (8.9)	64 (10.0)	
Grade (%)			<0.001			0.947
Well-differentiated	1286 (49.3)	198 (31.0)		197 (30.9)	198 (31.0)	
Moderately differentiated	985 (37.8)	251 (39.3)		256 (40.1)	251 (39.3)	
Poorly differentiated	301 (11.5)	163 (25.5)		156 (24.5)	163 (25.5)	
Undifferentiated	35 (1.3)	26 (4.1)		29 (4.5)	26 (4.1)	
T			<0.001			0.016
T1	2576 (98.8)	586 (91.8)		608 (95.3)	586 (91.8)	
T2	31 (1.2)	52 (8.2)		30 (4.7)	52 (8.2)	
Surgery			<0.001			0.61
Radical hysterectomy	2275 (48.9)	366 (57.4)		376 (58.9)	366 (57.4)	
Radiotherapy	1332 (51.1)	272 (42.6)		262 (41.1)	272 (42.6)	
Tumor size (cm)			<0.001			0.829
<=1.0	919 (35.3)	183 (28.7)		181 (28.4)	183 (28.7)	
1.0-2.0	790 (30.3)	196 (30.7)		183 (28.7)	196 (30.7)	
2.0-4.0	712 (27.3)	175 (27.4)		184 (28.8)	175 (27.4)	
>4.0	186 (7.1)	84 (13.2)		90 (14.1)	84 (13.2)	

RT, Radiotherapy; PSM, Propensity score matching; AC, Adenocarcinoma; NECC, Neuroendocrine carcinoma; CCA, Clear cell adenocarcinoma; EC, Endometrioid carcinoma.

**Table 5:** Baseline characteristics of patients with pT1-T2aN0M0 cervical adenocarcinoma in the validation cohort.

Characteristic	No. of patients before PSM (%)			No. of patients after PSM (%)		
	No RT (n = 1146)	RT (n = 245)	P-value	No RT (n = 245)	RT (n = 245)	P-value
Age (years)			<0.001			0.771
<=50	837 (73.0)	137 (55.9)		135 (55.1)	137 (55.9)	
51-64	237 (20.7)	65 (26.5)		61 (24.9)	65 (26.5)	
>=65	72 (6.3)	43 (17.6)		49 (20.0)	43 (17.6)	
Marital status			0.613			0.927
Single	434 (37.9)	97 (39.6)		99 (40.4)	97 (39.6)	
Married	712 (62.1)	148 (60.4)		146 (59.6)	148 (60.4)	
Histology			<0.001			0.966
AC	1033 (90.1)	180 (73.5)		184 (75.1)	180 (73.5)	
NEC	12 (1.0)	4 (1.6)		4 (1.6)	4 (1.6)	
EC	79 (6.9)	40 (16.3)		39 (15.9)	40 (16.3)	
CCA	22 (1.9)	21 (8.6)		18 (7.3)	21 (8.6)	
Race (%)			0.206			0.77
White	987 (86.1)	205 (83.7)		210 (85.7)	205 (83.7)	
Black	45 (3.9)	16 (6.5)		13 (5.3)	16 (6.5)	
Other	114 (9.9)	24 (9.8)		22 (9.0)	24 (9.8)	
Grade (%)			<0.001			0.985
Well-differentiated	545 (47.6)	59 (24.1)		58 (23.7)	59 (24.1)	
Moderatelydifferentiated	435 (38.0)	95 (38.8)		96 (39.2)	95 (38.8)	
Poorly differentiated	147 (12.8)	76 (31.0)		78 (31.8)	76 (31.0)	
Undifferentiated	19 (1.7)	15 (6.1)		13 (5.3)	15 (6.1)	
T			<0.001			0.015
T1	1133 (98.9)	216 (88.2)		232 (94.7)	216(88.2)	
T2	13 (1.1)	29 (11.8)		13 (5.3)	29 (11.8)	
Surgery			<0.001			0.85
Radical hysterectomy	574 (50.1)	160 (65.3)		157(64.1)	160 (65.3)	
Radiotherapy	572 (49.9)	85 (34.7)		88 (35.9)	85 (34.7)	
Tumor size (cm)			0.002			0.739
<=1.0	421 (36.7)	74 (30.2)		66 (26.9)	74 (30.2)	
1.0-2.0	339 (29.6)	68 (27.8)		72 (29.4)	68 (27.8)	
2.0-4.0	308 (26.9)	68 (27.8)		76 (31.0)	68 (27.8)	
>4.0	78 (6.8)	35 (14.3)		31 (12.7)	35 (14.3)	

RT, Radiotherapy; PSM, Propensity score matching; AC, Adenocarcinoma; NECC, Neuroendocrine carcinoma; CCA, Clear cell adenocarcinoma; EC, Endometrioid carcinoma.

## Discussion

In the present study, we investigated a cohort of 4636 patients with early postoperative cervical adenocarcinoma and constructed and internally validated 1-, 3- and 5-year nomograms to predict patient OS. This nomogram can be personalized to accurately predict the prognosis of the disease and facilitate clinical decision-making. Using the SEER database, some previous studies constructed nomograms of early forms of postoperative cancers, including non-small cell lung cancer (24), triple-negative breast cancer (25) and esophageal cancer (26) to predict patient outcomes. To date, no specific nomogram has been reported for surgically resected early-stage cervical adenocarcinoma.

In this study, RSF analysis revealed that several subgroup variables, including age (>=65 years), NECC, G3, G4 and T2 contributed the most to mortality. NECC is a rare but highly malignant cervical tumor with a poor prognosis. In a previous study, Intaraphet et al. suggested that adjuvant chemotherapy may provide a survival benefit for patients with early-stage small cell neuroendocrine carcinoma of the uterine cervix (SNECC) when treated with surgery (27). In another study, Lee et al. also recommended that systemic chemotherapy should be used as part of the initial treatment of SNECC (28). However, Prodromidou et al. reviewed the literature and concluded that for large cell cervical neuroendocrine carcinoma, surgery and lymphadenectomy had a significant effect on survival while chemotherapy and radiotherapy did not appear to have a significant effect on prognosis (29). By applying multivariate Cox analysis, we identified age, race, histology, grade, tumor size and radiotherapy as independent prognostic factors in patients with early-stage surgical resection. RSF analysis also demonstrated the importance of different factors to prognosis, including grade, age and tumor size. In the CSF model, the prognostic importance of variables is in order of grade, radiotherapy, chemotherapy, tumor Size, age, etc. Previous studies of the prognostic factors related to cervical adenocarcinoma have focused on lymph node status, tumor size, tumor grade, depth of cervical invasion, lymphovascular invasion (LVSI) and parametrial invasion (30–35). However, there is a notable lack of prospective studies featuring large sample sizes. It is worth noting that with the release of the new FIGO staging system in 2018, cervical cancer patients with nodal disease detected by imaging are now included in stage IIIC, thus implying that the best treatment for these patients is concurrent chemoradiotherapy, rather than surgery.

In this study, we focused on patients with early-stage cervical adenocarcinoma without nodal metastasis and parametrial invasion after surgery. Notably, tumor size (>2.0cm) was not found to be an independent prognostic factor for early-stage cervical adenocarcinoma in our study. However, previous studies suggested that tumor size was associated with early cervical adenocarcinoma metastasis and recurrence (36–38). In our cohort, there were missing values for tumor size in some patients; although we used the random forest algorithm to fill in these missing values, this practice may have interfered with the results we obtained. Therefore, whether tumor size (>2.0cm) is an independent prognostic factor for cervical adenocarcinoma still needs further verification.

In addition, we also constructed a nomogram that combined the risk predictors identified by our analysis. Calibration curves showed excellent consistency between predicted survival and actual observations. Moreover, both the nomogram C-index and ROC curve analysis revealed that the nomogram had a good predictive effect. However, the robustness of our model can be further improved if risk factors such as potential serum biomarkers and vascular invasion are included.

Currently, adjuvant therapy for cervical cancer, especially adenocarcinoma with intermediate-risk factors is still debatable. According to NCCN guidelines, patients with cervical cancer and negative nodes, margins and parametrium after surgery, should receive pelvic EBRT with or without concurrent platinum-containing chemotherapy if the patient meets the risk factors of the Sedlis criteria (i.e., primary tumor size, stromal invasion, and/or LVSI) (11). However, to the best of our knowledge, the current clinical evidence to support this recommendation remains insufficient. Cao et al. believe that patients with intermediate-risk cervical cancer (according to Sedlis criteria) can still achieve good survival with radical hysterectomy alone without adjuvant therapy (39). Some researchers have also analyzed patients with FIGO 2009 IB1 cervical cancer post-surgery and found that intermediate risk patients who did not receive adjuvant therapy after surgery did not show an increased recurrence rate and LVSI was the only risk factor affecting PFS and DSS (40). Okazawa et al. suggested that postoperative concurrent chemoradiotherapy can improve the prognosis of patients with FIGO stage IB1-IIB cervical cancer in high-risk groups and patients with two or more intermediate-risk factors (41). For early-stage cervical clear cell adenocarcinoma with moderate risk factors, Liu et al. concluded that radiotherapy did not improve prognosis; therefore, radical surgery alone was recommended for early-stage patients without high-risk factors (42). In another study, Glaze et al. analyzed 166 patients with cervical adenocarcinoma. Univariate analysis showed that premenopausal status, tumor size, first-line chemotherapy, LVSI, rare histological subtypes, FIGO stage and the receipt of second-line treatment were significantly associated with a lower OS. However, multivariate analysis further showed that only FIGO stage was an independent factor (43).

In this study, postoperative cervical adenocarcinoma patients with pT1-2aN0M0 were divided into three subgroups based on risk scores calculated from nomograms. The effects of different adjuvant treatments on survival were compared in each subgroup. For patients in the low-risk group, adjuvant chemotherapy had a worse prognosis than surgery alone. In the moderate- and high-risk groups, adjuvant chemoradiotherapy did not benefit patients, thus implying that a comprehensive risk assessment should be made when considering the therapy/benefit ratio of adjuvants. Because of the difficulties associated with randomized controlled trial (RCT), to compare therapeutic outcomes between individuals who have received a particular treatment regimen to those who have not. However, PSM analysis of patients can be used as a substitute for a RCT, at least in part (44). PSM has been proven to reduce the bias caused by confounding variables; thus, in our study, we were able to use PSM to produce reliable results and conclusions. In the present study, survival analysis showed that adjuvant chemoradiotherapy not only failed to improve OS; it was also associated with a worse prognosis. Then, we used the PSM method to further conduct survival analysis. Prior to PSM, high-risk groups, such as grade, histology and T stage were more inclined to receive adjuvant chemoradiotherapy. After matching, survival analysis found no significant difference between adjuvant radiotherapy and surgery alone in the validation cohort. In the training cohort, adjuvant radiotherapy was associated with a worse prognosis than surgery alone. Previous studies also found that radiation therapy only plays a limited role in early-stage clear cell adenocarcinoma of the uterine cervix with risk factors (42, 45). The results of our present analysis suggest that postoperative adjuvant chemoradiotherapy does not provide a survival benefit for patients with early-stage pT1-T2aN0M0 cervical adenocarcinoma.

To our knowledge, this is the largest study (4636 cases) to compare the prognosis of patients with early postoperative cervical adenocarcinoma. However, there are some limitations that need to be considered. First, we lacked information related to the prognosis after surgical resection, including potential biological biomarkers, the depth of cervical invasion, LVSI and HPV infection. Second, our training and validation sets were created by the same database and were limited to the United States; thus, datasets from other countries are also required for external validation to improve the robustness of our model. Third, the SEER database has not yet collected tumor recurrence data, so it is impossible to evaluate the progression free survival, and the effectiveness of rescue therapy. Finally, our study was retrospective; inherent selection biases are known to exist in any retrospective study. Therefore, further prospective clinical studies are still needed for further verification.

## Conclusion

We identified several clinicopathological variables that were independent risk factors for pT1-2aN0M0 stage cervical adenocarcinoma, including grade, age, T stage and histology. Postoperative patients did not benefit from adjuvant chemoradiotherapy. Whether patients with positive lymph nodes and incisional margins can benefit from adjuvant therapy, we are prepared to further present it in the follow-up study. Our findings may provide useful references and guidelines for future treatment decisions.

## Data availability statement

The original contributions presented in the study are included in the article/supplementary material. Further inquiries can be directed to the corresponding author.

## Ethics statement

Ethical review and approval was not required for the study on human participants in accordance with the local legislation and institutional requirements. Written informed consent for participation was not required for this study in accordance with the national legislation and the institutional requirements.

## Author contributions

Conception and design: DO. Development of methodology: DO. Extracted and analyzed the data: DO, MS, and LL. Writing, review, and/or revision of the manuscript: All authors. All authors read and approved the final manuscript.

## Funding

This work was supported by grants from Taizhou "500 Elite Plan" high-level talent project.

## Acknowledgments

We are very grateful to the staff in Surveillance, Epidemiology, and End Results Program (SEER) for their kind work in data collection and delivery. And we thank Professor Xueqiong Zhu at the Department of Obstetrics and Gynecology, The Second Affiliated Hospital of Wenzhou Medical Universit, China, for guiding us.

## Conflict of interest

The authors declare that the research was conducted in the absence of any commercial or financial relationships that could be construed as a potential conflict of interest.

## Publisher’s note

All claims expressed in this article are solely those of the authors and do not necessarily represent those of their affiliated organizations, or those of the publisher, the editors and the reviewers. Any product that may be evaluated in this article, or claim that may be made by its manufacturer, is not guaranteed or endorsed by the publisher.
